# Integrative Proteomics and Genome-Scale Modeling Elucidate Metabolic Flux Shifts in *Chlamydomonas reinhardtii* CC-4414 Under Light Stress

**DOI:** 10.3390/ijms27188383

**Published:** 2026-09-20

**Authors:** Kittichai Yosdee, Vichugorn Wattayagorn, Yuke He, Wanida Pan-utai, Solange I. Mussatto, Pramote Chumnanpuen, Wanwipa Vongsangnak

**Affiliations:** 1 Kasetsart University International College, Kasetsart University, Bangkok 10900, Thailand; kittichai.yo@ku.th (K.Y.); vichugorn.wat@ku.th (V.W.); 2 KUSynBio Special Research Incubator Unit, Kasetsart University International College (KUIC), Kasetsart University, Bangkok 10900, Thailand; 3 Interdisciplinary Graduate Program in Bioscience, Faculty of Science, Kasetsart University, Bangkok 10900, Thailand; 4 Department of Applied Microbiology, Institute of Food Research and Product Development, Kasetsart University, Bangkok 10900, Thailand; 5 Department of Biotechnology and Biomedicine, Technical University of Denmark, Søltofts Plads, Building 223, 2800 Kongens Lyngby, Denmark; smussatto@dtu.dk; 6 Department of Zoology, Faculty of Science, Kasetsart University, Bangkok 10900, Thailand

**Keywords:** *Chlamydomonas reinhardtii* CC-4414, genome-scale metabolic model (GSMM), metabolic flux analysis, light stress, proteomics integration, metabolic reprogramming

## Abstract

The green microalga *Chlamydomonas reinhardtii* holds substantial promise as a photosynthetic cell factory for sustainable bioproduction, yet strain-specific metabolic responses to environmental stress remain underexplored. Here, we reconstructed a genome-scale metabolic network (GSMN) of the light-tolerant strain *C. reinhardtii* CC-4414 by combining de novo genome assembly with integrative proteomics. The resulting model, designated *iYH2021*, comprises 2021 genes, 2601 reactions, and 2089 metabolites, representing the first genome-scale metabolic reconstruction reported for this strain. To elucidate light-driven metabolic remodeling, we integrated quantitative proteomic data from high-light (HL) and low-light (LL) conditions into the model using the iMAT algorithm and performed metabolic flux analysis (MFA). Because these context-specific networks integrate only proteomically supported reactions, flux predictions were evaluated using an objective function that captures photon-handling capacity, providing a direct readout of how the photosynthetic apparatus reallocates flux under each condition. Our predictions revealed that HL conditions significantly enhanced fluxes through photosynthetic electron transport, the Calvin–Benson cycle, glycolysis, and the tricarboxylic acid (TCA) cycle, indicating a reallocation of carbon and energy metabolism toward light-driven pathways, while amino acid metabolism showed a mixed pattern of change. In contrast, LL conditions induced a marked upregulation of purine metabolism, suggesting accelerated nucleotide turnover as an adaptive response to light limitation. A complementary growth-rate analysis using the unconstrained network further indicated a higher maximum growth rate under HL than LL, consistent with the expected benefit of greater light availability. These condition-specific flux redistributions highlight the metabolic plasticity of *C. reinhardtii* CC-4414 and provide a systems-level understanding of how light intensity shapes carbon and energy allocation. The *iYH2021* model thus offers a robust predictive platform for guiding metabolic engineering strategies aimed at optimizing the production of biofuels and high-value bioproducts in this industrially relevant microalga under stress conditions. These findings represent model-derived predictions and have not yet been experimentally validated. We emphasize that this predictive platform requires experimental validation before its use in guiding metabolic engineering decisions.

## 1. Introduction

The global demand for renewable energy sources, bioplastics, and high-value biobased products has significantly increased in an effort to mitigate environmental problems [1,2]. Within the framework of sustainable biological platforms, microalgae are unicellular eukaryotic organisms distributed across diverse aquatic ecosystems and serve as highly potent cellular factories for the synthesis of valuable compounds such as the pigments β-carotene and astaxanthin and the polymers alginate, carrageenan, and agar for food products [1,2]. Moreover, microalgae grow rapidly and require only sunlight, water, and CO_2_, making them highly promising cell factories for sustainable bioproduction [3,4]. However, in large-scale microalgal cultivation, fluctuating light conditions—ranging from excessive high-light (HL) that induces photoinhibition and reactive oxygen species (ROS) accumulation, to low-light (LL) that limits photosynthetic energy supply—pose significant challenges for biomass and bioproduct yields. Understanding how photosynthetic organisms perceive and adapt to such light fluctuations is therefore essential for optimizing their performance as sustainable cell factories.

The green microalga *Chlamydomonas reinhardtii* has emerged as a powerful model system for studying photosynthesis, cilia biogenesis, and stress responses [3,4]. *C. reinhardtii* exhibits significantly high photosynthetic carbon dioxide fixation and enables rapid proliferation even under environmental stress conditions. Furthermore, this microalga can be genetically or metabolically engineered to enhance the production of lipids, bio-hydrogen, and specialized biochemicals [5]. The availability of a high-quality reference genome, initially published by Merchant et al. (2007) [6] and subsequently refined, has further accelerated research on this alga. Nevertheless, most laboratory strains of *C. reinhardtii* have been maintained under controlled conditions for decades, potentially losing their robustness to environmental fluctuations. Consequently, these strains often exhibit limited tolerance to high light intensities and other abiotic stresses, constraining their applicability in outdoor industrial cultivation [7,8]. To overcome these limitations, attention has turned to natural strains that retain stress-resilience traits, offering superior performance under fluctuating environmental conditions.

Excess light poses a fundamental physiological challenge for photosynthetic organisms, and *C. reinhardtii* has evolved a layered set of mechanisms to manage it. At the level of acute photodamage, light energy absorbed in excess of photosynthetic capacity drives the formation of reactive oxygen species (ROS), including singlet oxygen generated at photosystem II (PSII) and superoxide/hydrogen peroxide generated via the Mehler reaction at photosystem I; unless dissipated, this damages the PSII D1 reaction-centre protein (photoinhibition) and, more broadly, membrane lipids, proteins, and nucleic acids. To limit this damage, short-term (seconds to minutes) photoprotective responses are induced, most notably non-photochemical quenching (NPQ) of excess excitation energy as heat via the LHCSR-dependent xanthophyll cycle, together with state transitions that redistribute light-harvesting antennae between the two photosystems and cyclic electron flow around PSI that relieves pressure on the downstream electron-transport chain. Over longer timescales (hours-to-days), photoacclimation further adjusts the antenna size and pigment composition of the photosynthetic apparatus and the relative abundance of PSI and PSII, reducing light-harvesting capacity under sustained high light. Under low light, the converse acclimation strategy predominates: cells typically increase antenna size and chlorophyll content per cell to maximize capture of the limited available light, at the cost of reduced protection should light intensity increase abruptly. These general mechanisms, and how they are engaged differently by natural strains with contrasting light tolerance, are discussed in more detail below and in the source proteomics study underlying the present model [8].

A particularly promising strain is *C. reinhardtii* CC-4414 (also known as DN2), a light-tolerant originally isolated from a tropical environment. Unlike conventional laboratory strains, CC-4414 exhibits remarkable resilience to high light stress through multiple protective mechanisms, including palmelloid formation, cell aggregation, and efficient ROS management. Suwannachuen et al. (2023) [8] demonstrated that the light-tolerant strain CC-4414 managed ROS more effectively than the sensitive strain CC-2344, with proteomic data revealing cellular modifications toward membrane proteins and the induction of palmelloid structures and cell aggregation to shield cells from excessive light. These morphological and physiological adaptations enable CC-4414 to maintain photosynthetic activity and cell viability under HL conditions that would severely impair standard strains. Moreover, CC-4414 demonstrates exceptional phenotypic plasticity and metabolic resilience against chemical stressors compared to standard laboratory strains. Previous studies have shown that CC-4414 possesses a remarkable capacity to adapt and recover from initial chemical inhibitions, such as zinc oxide nanoparticles (ZnO NPs) [7]. Collectively, these traits position CC-4414 as an ideal candidate for investigating the molecular and metabolic basis of light stress tolerance in microalgae, with direct relevance to industrial-scale cultivation under fluctuating light regimes.

To fully exploit the biotechnological potential of *C. reinhardtii* for targeted bioproduct synthesis, advanced systems biology approaches are indispensable. The highly dynamic nature of photosynthetic networks in response to fluctuating environmental stimuli necessitates robust computational frameworks to decipher complex biochemical processes. Genome-scale metabolic models (GEMs) provide powerful predictive frameworks to understand cell phenotypes and guide metabolic engineering in silico [9,10]. By integrating genomic, transcriptomic, and proteomic data, GEMs provide a platform for omics data analysis and phenotype prediction. For *C. reinhardtii*, several GEMs have been reconstructed over the past decade. Chang et al. (2011) [11] reconstructed the first genome-scale metabolic network for this alga and devised a novel light-modeling approach that enables quantitative growth prediction for a given light source. Subsequently, Dal’Molin et al. (2011) [12] developed AlgaGEM, a genome-scale metabolic reconstruction of algae based on the *C. reinhardtii* genome. The most comprehensive reconstruction to date is *iCre1355*, developed by Imam et al. (2015) [13], which represents a major advance over previous models both in content and predictive power. *iCre1355* encompasses a broad range of metabolic functions encoded across the nuclear, chloroplast, and mitochondrial genomes, accounting for 1355 genes, 2394 reactions, and 1133 metabolites. This experimentally validated reconstruction has been used to analyze high-resolution time-series transcriptomics data, enabling accurate prediction of growth rates and the temporal response of different growth-associated pathways to increased light intensity.

Despite the availability of these GEMs for *C. reinhardtii*, a strain-specific model that incorporates the distinct metabolic traits of *C. reinhardtii* CC-4414 remains conspicuously absent. First, existing models are primarily based on reference strains (such as CC-125 or CC-1690), which lack the stress-tolerance traits of natural strains, such as CC-4414. Second, the integration of condition-specific proteomic data into GEMs for predicting metabolic flux shifts under light stress has not been systematically explored in this strain. While recent advances have demonstrated the potential of context-specific genome-scale modeling to interpret metabolic phenotypes under various conditions [14], a strain-specific model that captures the unique metabolic traits and stress-tolerance mechanisms of *C. reinhardtii* CC-4414 remains unavailable. Consequently, integrating these specific photoprotective, antioxidant, and stress-tolerance mechanisms into a newly reconstructed GEM will facilitate a more precise prediction of metabolic fluxes, ultimately guiding successful in silico metabolic interventions for enhanced strain performance.

To address these gaps, this study aimed to (1) reconstruct a genome-scale metabolic network (GSMN) of *C. reinhardtii* CC-4414 based on its de novo genome assembly; (2) integrate quantitative proteomic data from high-light (HL) and low-light (LL) conditions into the model using the iMAT algorithm; (3) perform metabolic flux analysis (MFA) to elucidate condition-specific flux redistributions; and (4) identify key metabolic pathways and reactions that underpin the light-stress adaptation of this strain. By linking proteomic profiles to metabolic flux predictions, this integrative approach provides a systems-level understanding of how *C. reinhardtii* CC-4414 adjusts its carbon and energy metabolism in response to light stress. The resulting model, *iYH2021*, not only fills the gap in strain-specific GEMs for CC-4414 but also serves as a predictive platform for guiding metabolic engineering strategies aimed at optimizing the production of biofuels and high-value bioproducts under industrial light regimes. This work thus contributes to the broader field of algal stress physiology and supports the development of sustainable microalgal biorefineries.

Based on the reported photoprotective phenotype of CC-4414 including palmelloid formation, cell aggregation, and efficient ROS management. High-light (HL) exposure would preferentially redirect fluxes toward photosynthetic electron transport, the Calvin–Benson cycle, and downstream carbon/energy-generating pathways (glycolysis and the TCA cycle) to support rapid growth and photoprotection, whereas low-light (LL) exposure would constrain these energy-generating pathways and instead be associated with a shift toward maintenance-related and stress-responsive metabolism. This hypothesis was tested by comparing iMAT-derived, condition-specific flux distributions between the HL- and LL-associated models.

## 2. Results

### 2.1. Genome Assembly and Annotation

#### 2.1.1. Genome Assembly Quality

The de novo genome assembly of *C. reinhardtii* CC-4414 evaluated using QUAST v5.3.0 yielded a total assembly length of 94.02 Mb, distributed across 12,924 contigs (≥500 bp), with a genomic GC content of 64.30% (Table 1). The assembly achieved an N50 contig length of 14,926 bp and an L50 count of 1777, indicating a high degree of sequence continuity suitable for downstream metabolic network reconstruction. Genome completeness assessed by BUSCO v6.0.0 against the chlorophyta_odb10 lineage dataset (*n* = 62) revealed 89.1% complete BUSCOs (including 0.8% duplicated), 7.1% fragmented BUSCOs, and 3.8% missing BUSCOs, confirming successful recovery of the majority of core conserved genes.

#### 2.1.2. Functional Annotation and Taxonomic Classification

**Figure 1:**
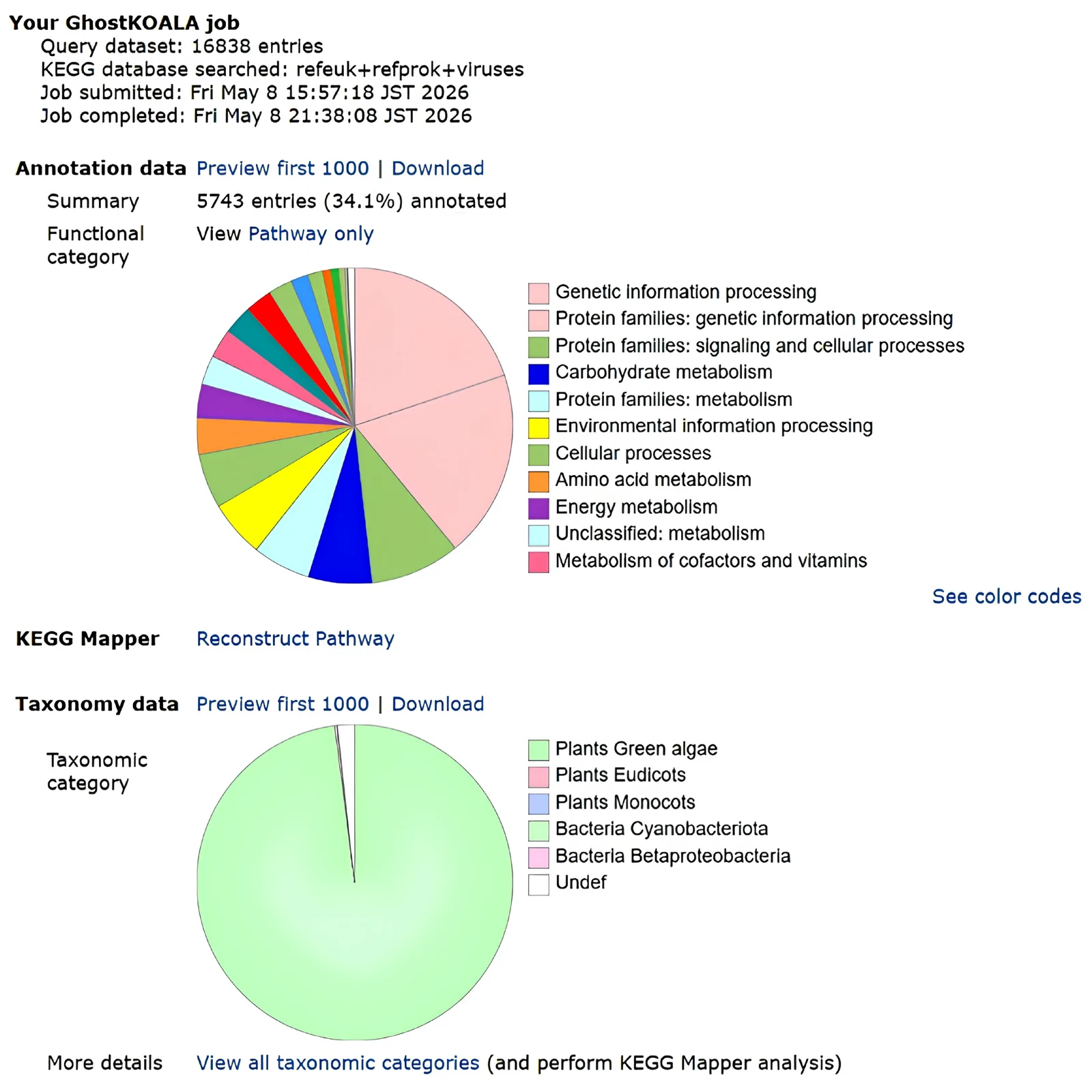
Functional annotation and taxonomic classification of predicted proteins from *C. reinhardtii* CC-4414 using GhostKOALA against the KEGG database.

Functional annotation of 16,838 predicted protein sequences using GhostKOALA successfully assigned 5743 sequences (34.1%) to specific KEGG pathways. Taxonomic distribution analysis confirmed the high purity of the assembled genome, with the majority of predicted proteins aligning with chlorophyte lineages, indicating minimal microbial contamination. KEGG functional classification revealed that a substantial proportion of annotated genes were associated with genetic information processing and core metabolic infrastructure, including carbohydrate metabolism (21%), amino acid metabolism (16%), and energy metabolism (10%) (Figure 1). Quantitative contamination-relevant metrics are as follows: BUSCO analysis (chlorophyta_odb10 lineage dataset) showed 89.1% completeness with a duplication rate of 0.7%, and QUAST reported an overall genomic GC content of 64.30%, both consistent with expected Chlamydomonas genomic characteristics (see Section 3 for interpretation and methodological concerns). The total length of all assembled contigs before filtering was 103.02 Mb; the 94.02 Mb value reported in Section 2.1.1 represents the assembly statistics after removing short contigs (<500 bp) per the QUAST filtering criterion.

### 2.2. Characteristics of the Reconstructed C. reinhardtii CC-4414 GSMN

#### 2.2.1. Model Composition

After manual curation and integration, the reconstructed genome-scale metabolic network of *C. reinhardtii* CC-4414, designated *iYH2021*, comprised 2021 genes, 2601 reactions, and 2089 metabolites distributed across 10 cellular compartments (Table 2). Compared to the template model *iCre1355* [13], which contains 1355 genes, 2394 reactions, and 1845 metabolites, *iYH2021* represents a substantial expansion in both genomic coverage and metabolic reaction content, reflecting the strain-specific gene complement of CC-4414. It should be noted that part of this apparent expansion in gene and reaction content likely reflects differences in the gene-prediction pipeline and genome assembly strategy used (AUGUSTUS/SPAdes-based draft assembly versus the curated JGI v5.5 reference used for *iCre1355*) rather than the addition of new metabolic functions per se; only 34.1% of the 16,838 predicted proteins could be assigned to a KEGG pathway (Section 2.1.2), indicating that a substantial fraction of the additional gene content is functionally uncharacterized or non-metabolic. This concern is discussed further in Section 3.

#### 2.2.2. Pathway Coverage

**Figure 2:**
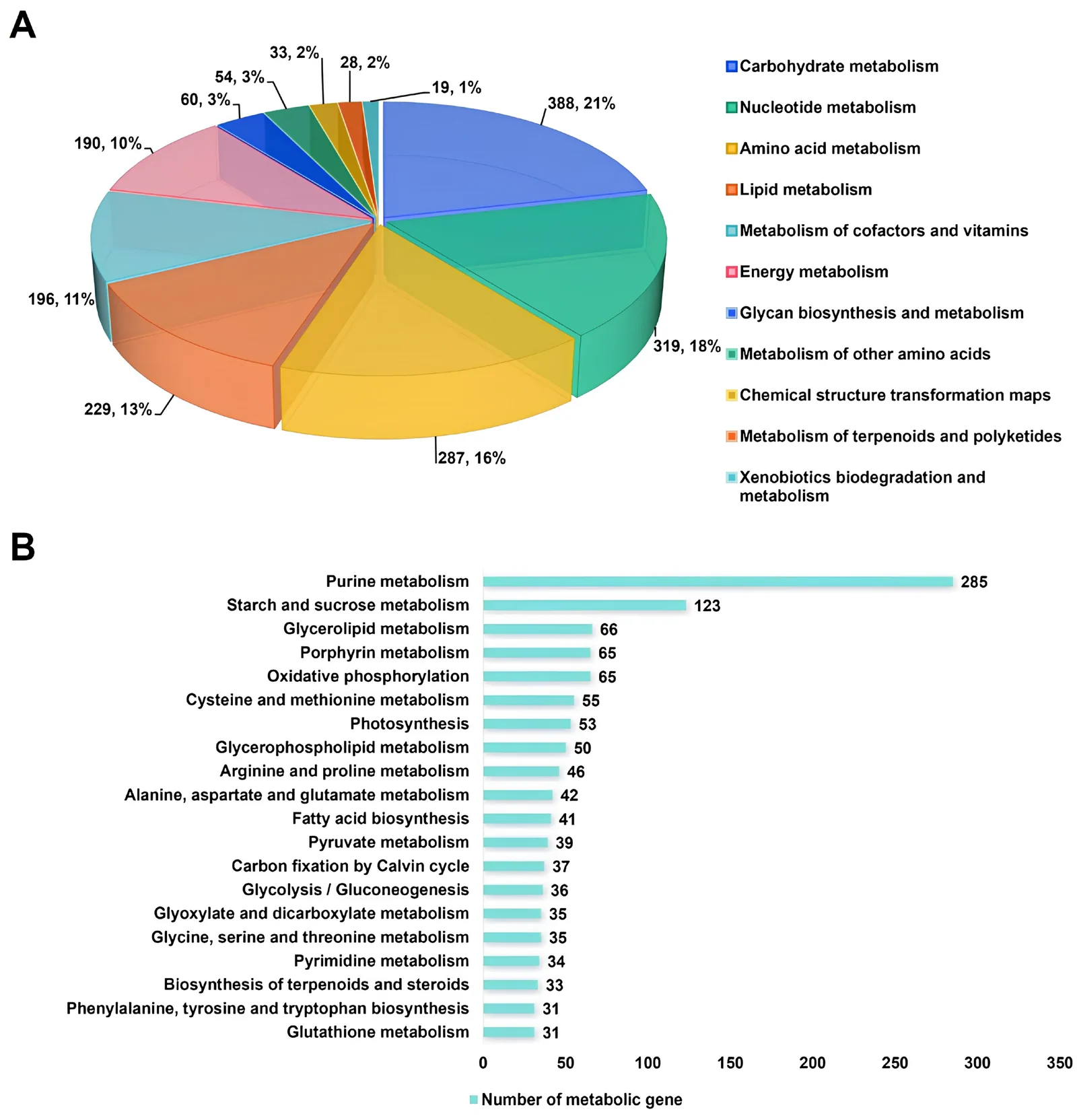
Functional classification of metabolic genes represented in the GSMN of *C. reinhardtii* CC-4414. (**A**) Overview of major metabolic functional categories. (**B**) Top 20 sub-metabolic functional categories showing the most represented metabolic genes across pathways. Note: Genes assigned to multiple sub-metabolic categories were counted only once in the major metabolic categories.

Functional classification of metabolic genes according to the KEGG database revealed that the largest proportion of genes was associated with carbohydrate metabolism (388 genes, 21%), followed by nucleotide metabolism (319 genes, 18%) and amino acid metabolism (287 genes, 16%) (Figure 2A). Lipid metabolism (229 genes, 13%), metabolism of cofactors and vitamins (196 genes, 11%), and energy metabolism (190 genes, 10%) were also highly represented. Smaller gene sets were assigned to glycan biosynthesis and metabolism (60 genes, 3%), metabolism of other amino acids (54 genes, 3%), terpenoid and polyketide metabolism (28 genes, 2%), and xenobiotic biodegradation (19 genes, 1%) (Figure 2A). At the sub-pathway level (Figure 2B), purine metabolism contained the highest number of genes (285 genes), followed by starch and sucrose metabolism (123 genes). Pathways related to lipid and energy metabolism were also highly enriched, including glycerolipid metabolism (66 genes), porphyrin metabolism (65 genes), oxidative phosphorylation (65 genes), and glycerophospholipid metabolism (50 genes). Key pathways involved in carbon assimilation and central metabolism, including photosynthesis (53 genes), the Calvin cycle (37 genes), glycolysis/gluconeogenesis (36 genes), pyruvate metabolism (39 genes), and the glyoxylate cycle (35 genes), were also well represented.

To place these pathway proportions in context, we compared the reaction-level subsystem distribution of *iYH2021* directly against the *iCre1355* template using the reaction annotations in both model files (rather than gene-level KEGG classification, which is not available for *iCre1355*). This shows a broadly similar functional profile between the two models: lipid metabolism (24.2% of reactions in *iCre1355* versus 21.9% in *iYH2021*), amino acid metabolism (13.7% versus 12.8%), carbohydrate metabolism (6.5% versus 7.1%), and cofactor/vitamin metabolism (7.2% versus 7.0%) each account for a similar proportion of reactions in both models. The clearest difference is in nucleotide metabolism, which accounts for a larger share of reactions in *iYH2021* than in *iCre1355* (12.6% versus 8.6%), consistent with the expanded purine-related gene/reaction content noted in Section 2.2.1; terpenoid/polyketide metabolism is also proportionally larger in *iYH2021* (3.3% versus 1.4%). Overall, this comparison indicates that the additional genes and reactions in *iYH2021* are distributed across functional categories in a pattern broadly consistent with the *iCre1355* template, rather than being concentrated in a single, unusual category.

### 2.3. Metabolic Flux Analysis Under Varying Environmental Light Conditions

#### 2.3.1. Context-Specific Model Reconstruction

To reconstruct context-specific metabolic states of *C. reinhardtii* CC-4414 under different light conditions, quantitative proteomic data from HL and LL conditions [8] were integrated into the *iYH2021* model using the iMAT algorithm [15]. The integration successfully generated two condition-specific models representing the metabolic networks active under HL and LL conditions, respectively.

#### 2.3.2. Flux Distribution Under HL and LL Conditions

**Figure 3:**
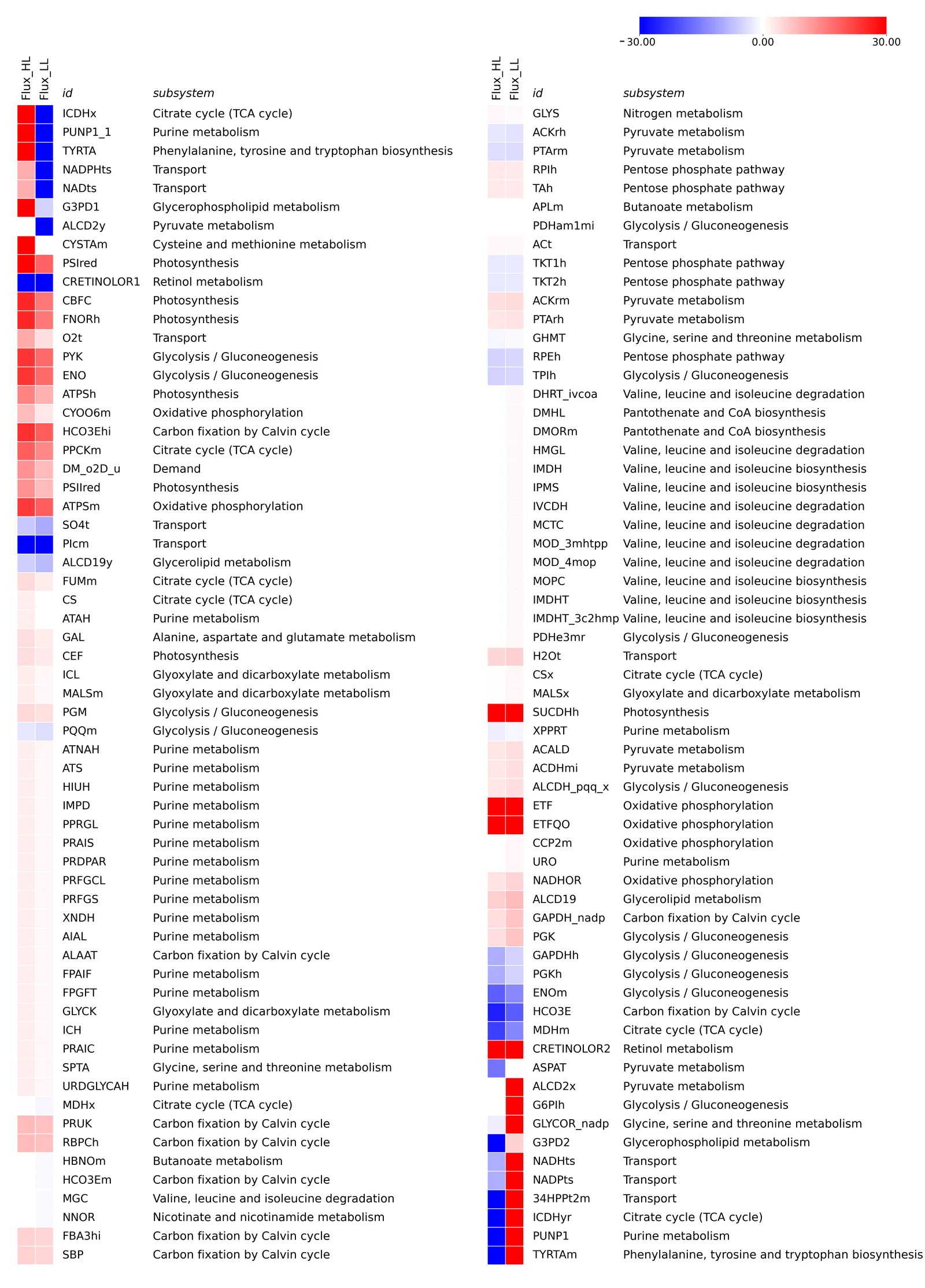
Heatmap of predicted relative metabolic flux distribution under high light (${\mathrm{Flux}}_{\mathrm{HL}}$) and low light (${\mathrm{Flux}}_{\mathrm{LL}}$) conditions across reactions and subsystems. The color scale at the top indicates relative flux values ranging from −30.00 (blue) to +30.00 (red), centered at 0.00 (white). Each row represents a specific reaction or subsystem, accompanied by its corresponding reaction ID and subsystem name. Red-shaded reactions indicate higher flux intensity, whereas blue-shaded reactions represent lower or reversed flux under the specified condition.

**Figure 4:**
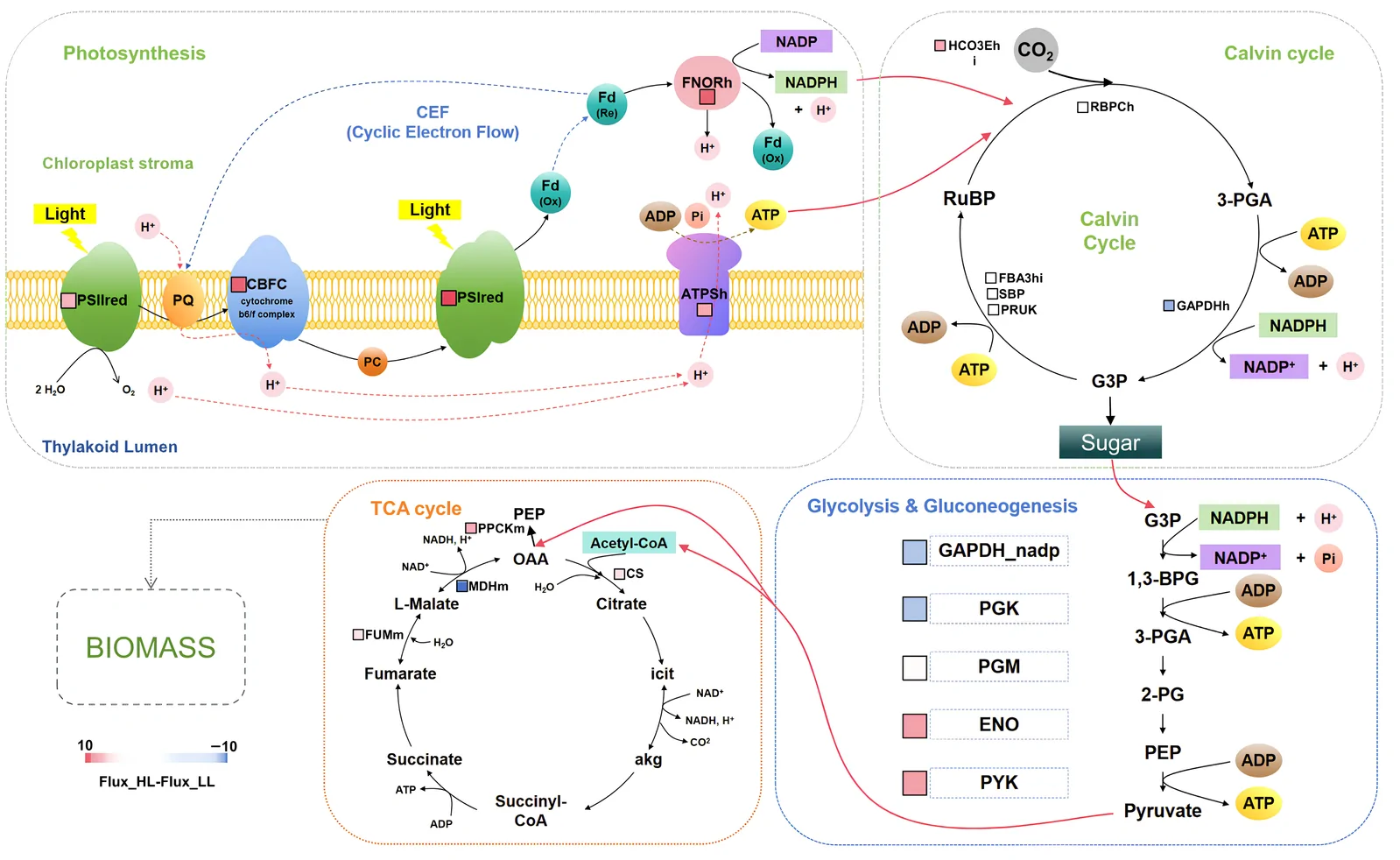
Comparative metabolic flux distribution of *C. reinhardtii* under HL and LL conditions. Schematic integration of predicted metabolic fluxes into core metabolic pathways, including photosynthesis (thylakoid membrane), the Calvin-Benson cycle (chloroplast stroma), cytosolic glycolysis/gluconeogenesis, and the mitochondrial TCA cycle. Colored boxes adjacent to reaction labels indicate the relative flux differences (Flux_HL- Flux_LL), ranging from +10 (red, up-regulated under HL) to −10 (blue, down-regulated under HL/up-regulated under LL). Note: HCO_3_Ehi: bicarbonate transport RBPCh: RuBisCO PRUK: phosphoribulokinase FBA3hi: fructose-1,6-bisphosphate aldolase SBP: sedoheptulose-1,7-bisphosphatase PQ: plastoquinone. PC: Plastocyanin. Fd(Ox): Oxidized ferredoxin. Fd(Re): Reduced ferredoxin. RuBP: Ribulose 1,5-bisphosphate. 3-PGA: 3-Phosphoglyceric acid. G3P: glyceraldehyde 3-phosphate. 1,3-BPG: 1,3-Bisphosphoglyceric acid. 2-PG: 2-Phosphoglyceric acid. PEP: Phosphoenolpyruvic acid. OAA: Oxaloacetic acid. icit: isocitrate. akg: alpha-ketoglutarate.

Flux balance analysis (FBA) was performed on both context-specific models using the photon-handling objective described in Section 4.6. The predicted flux distributions revealed distinct metabolic reprogramming between HL and LL conditions, as summarized in Table 3 and visualized in Figure 3 and Figure 4. As FBA optimal solutions are generally non-unique, the flux distributions reported here correspond to one representative optimal solution for each condition-specific model. The robustness of these condition-specific differences was therefore assessed by flux variability analysis, presented in Section 2.3.3.

Under HL conditions, flux rates through the thylakoid membrane electron transport chain were markedly elevated compared to LL conditions. Specifically, fluxes through Photosystem II (PSIIred), Cytochrome b_6_f complex (CBFC), Photosystem I (PSIred), ATP synthase (ATPSh), and Ferredoxin-NADP^+^ reductase (FNORh) showed significant increases (Figure 3). This upregulation facilitated enhanced production of cellular energy (ATP) and reducing equivalents (NADPH).

The increased supply of ATP and NADPH under HL conditions drove enhanced carbon assimilation through the Calvin–Benson cycle, as evidenced by elevated fluxes in key reactions including HCO_3_Ehi (bicarbonate transport), RBPCh (RuBisCO), PRUK (phosphoribulokinase), FBA3hi (fructose-1,6-bisphosphate aldolase), and SBP (sedoheptulose-1,7-bisphosphatase) (Figure 4). These flux increases promoted the synthesis of glyceraldehyde 3-phosphate (G3P), which was subsequently routed through cytosolic glycolysis, exhibiting increased fluxes in reactions catalyzed by enolase (ENO) and pyruvate kinase (PYK), leading to enhanced pyruvate and acetyl-CoA production.

The increased glycolytic flux fed into the mitochondrial TCA cycle, with coordinated flux increases observed in citrate synthase (CS), isocitrate dehydrogenase (icit), fumarase (FUMm), and phosphoenolpyruvate carboxylase/kinase (PPCKm) (Figure 4). These flux redistributions indicated enhanced mitochondrial respiration to meet the elevated energy demands under HL conditions. We note that several TCA-cycle reactions (e.g., ICDHx) reached the ±1000 mmol gDW^−1^ h^−1^ flux bound in opposite directions between HL and LL in the underlying flux table, consistent with these reactions being constrained primarily by the wide bounds applied to protect central metabolism (Section 4.6) rather than by fully biologically resolved flux values. The description of the TCA cycle as “partially enhanced” under HL (Table 3) should therefore be interpreted with this concerns.

In contrast to HL conditions, purine metabolism exhibited an increasing trend under LL conditions (Figure 3 and Table 3). This upregulation suggests accelerated nucleotide turnover and potentially enhanced stress-signaling responses under light limitation. This trend is discussed critically in Section 3 with respect to its bioenergetic plausibility and possible numerical origin.

#### 2.3.3. Flux Variability Analysis

Of the 21 reactions tested, only two show non-overlapping (robust) FVA ranges between HL and LL: PGK (HL = 0–6, LL = 22.77–30.53; LL > HL) and, by construction, the objective PRISM_design_growth itself (HL = 1000, LL = 50). The remaining 19 reactions, including PSIred, CBFC, FNORh, PSIIred, and ATPSh (the core photosynthetic electron-transport reactions), show overlapping ranges: in each case the HL range spans from 0 up to a maximum that includes the corresponding LL value, which is itself fixed at a single point. On this basis, these photosynthesis-associated reactions cannot be considered robustly different between HL and LL. PUNP1_1, TYRTA, and ALAAT remain completely unconstrained (−1000 to 1000) in both conditions. A parallel full-network FVA (210 shared reactions) shows the same pattern at larger scale: 89 reactions (42%) are completely unconstrained in both conditions, and only 13 reactions show a robust, non-overlapping difference (3PG_pi_thr, ACCOAtm, ACKrm, ACtm, DM_photon646_h, DM_photon680_u, EX_photonVis_e, G3P_pi_thr, GAPDHh, PGK, PGKh, PRISM_design_growth, and PTArm). These results indicate that the majority of the condition-specific flux differences discussed in Table 3 and Figure 3 and Figure 4 are not uniquely determined by the model and proteomics constraints at the optimum, and should be interpreted as consistent with, rather than proof of, condition-specific regulation, pending independent experimental validation.

Carbon storage via starch and triacylglycerol (TAG) accumulation is a well-documented component of the stress response of *Chlamydomonas* and other microalgae, and both pathways are represented in *iYH2021*: starch synthase (STARCH300S) and a large set of acyl-CoA/phospholipid:diacylglycerol acyltransferase reactions (ACOADAGAT*, PLDAGAT*, covering multiple acyl-chain combinations) and triacylglycerol acylhydrolase reactions (TAGAH*) are all present in the model and contribute directly to the storage-lipid and starch components of the biomass function (Section 4.4). In the present analysis, however, none of these reactions appears among those with a detectable HL–LL flux difference (Table 3 and Table 4), indicating that the current iMAT-derived context-specific models do not predict a shift in carbon-storage allocation between the two conditions. This may reflect a genuine absence of differential regulation of these pathways under the specific HL/LL conditions and exposure duration sampled here, or may indicate that the corresponding genes lacked sufficient proteomic support to be distinguished from the intermediate-expression (unconstrained) category by the iMAT threshold procedure. Given the established importance of starch and lipid remodeling in light-stress responses in this species, we identify targeted proteomic or metabolomic quantification of starch- and TAG-pathway components under HL and LL as a priority for follow-up work.

### 2.4. Supplementary Theoretical Growth-Rate Analysis

Using the unrestricted genome-scale network with Biomass_Chlamy_mixo as the objective and EX_photonVis_e bounded at the HL and LL light capacities defined in Section 4.7, the estimated growth rates were HL = 0.151589 h^−1^ and LL = 0.121333 h^−1^ (HL/LL ratio = 1.249). The exchange-reaction sensitivity screen (Section 4.7) showed that predicted growth plateaus once photon uptake exceeds a lower bound of approximately −67, and that acetate (EX_ac_e) and CO_2_ (EX_co2_e) uptake have the largest effect on predicted biomass among all exchange reactions tested beyond this point. The two-dimensional sensitivity analysis over acetate and CO_2_ uptake bounds showed that these two substrates jointly, but non-additively, constrain growth in this mixotrophic model: for example, at an acetate bound of −2, raising the CO_2_ bound from −2 to −3 increased growth from 0.152 to 0.177, with no further increase from additional CO_2_ uptake; acetate was the stronger constraint overall, and its effect diminished once sufficient acetate was available (growth reached 0.213 at an acetate bound of −4.5, largely independent of the CO_2_ bound tested). Together, these results indicate that the LL condition used above (photon bound of −50) falls within a light-limited regime, whereas the HL condition (≥−80) falls within a carbon (acetate/CO_2_)-limited regime once light is no longer limiting; the HL-versus-LL growth-rate difference therefore reflects a genuine transition between these two limiting regimes rather than an arbitrary modeling choice. This growth-rate estimate is a distinct analysis, independent of the iMAT/proteomics-integrated flux-redistribution results presented in Section 2.3, and should not be conflated with them. The acetate and CO_2_ uptake bounds used as the default constraints in this analysis (−2 mmol gDW^−1^ h^−1^ each) were inherited directly from the iCre1355 mixotrophic template model and are not experimentally measured physiological uptake rates for CC-4414; in the original iCre1355 study, experimentally determined carbon uptake rates were used only when predicting quantitative growth under specific chemostat conditions. The absolute growth-rate values reported here should therefore be interpreted as illustrative of the model’s behavior under generic default constraints rather than validated, strain-specific predictions; the qualitative conclusion that growth transitions from a light-limited to a carbon-limited regime is independent of this concern, but the specific growth-rate values and the exact location of the transition would need to be re-examined using experimentally measured acetate/CO_2_ uptake rates for CC-4414, if available, before being used quantitatively.

## 3. Discussion

The integration of quantitative proteomic data with the genome-scale metabolic model *iYH2021* provided a systems-level view of how the light-tolerant strain *C. reinhardtii* CC-4414 redistributes metabolic flux under contrasting light regimes. Because the iMAT algorithm restricts each context-specific network to the subset of reactions supported by measurable protein abundance, the resulting HL- and LL-associated models retain only a minority of the full reaction set and do not support biomass synthesis without reintroducing the majority of the reactions removed during context-specific reconstruction. The flux distributions reported here are therefore best interpreted as reflecting differences in photon-handling capacity and internal carbon/energy redistribution between the two light conditions rather than differences in growth rate or biomass accumulation. To address growth directly, a complementary analysis was performed on the unrestricted genome-scale network, optimizing biomass production under HL- and LL-specific photon-uptake constraints; this indicated a higher theoretical maximum growth rate under HL than LL (0.152 versus 0.121 h^−1^), consistent with the general expectation that greater light availability supports higher growth capacity. This theoretical estimate should, however, be interpreted with reference to the culture behavior reported for this strain: under continuous high-light exposure, cell density does not increase monotonically but instead peaks within the first two days before declining, with visible pigment loss evident by day four even in this comparatively light-tolerant strain [8]. Net population growth under sustained high-light stress therefore reflects a balance between photosynthetic capacity and photodamage/degradative processes (protein turnover and autophagy) that are not represented in the core metabolic network, and the theoretical growth-rate comparison above is best regarded as an estimate of metabolic capacity rather than a validated prediction of observed growth.

Flux variability analysis (FVA) of the HL- and LL-context models indicated that most of the differences summarized in Table 3 are not uniquely determined by the available constraints: across the reactions examined, a substantial proportion carried a feasible flux range spanning the full bounds permitted by the model in both conditions, and only a small subset showed non-overlapping ranges between HL and LL. Among the core photosynthetic electron-transport reactions (photosystem I and II, cytochrome b6f, ferredoxin-NADP+ reductase, and ATP synthase), the HL-associated flux range was wide and included the corresponding LL value in each case, indicating that these reactions cannot be considered robustly different between conditions on the basis of the present analysis alone. Phosphoglycerate kinase was the clearest exception, showing a non-overlapping difference with higher flux under LL than HL. These results suggest that only a small number of central-metabolism reactions are distinguishable between the two light regimes once solution non-uniqueness is taken into account, and that pathway-level statements about enhanced photosynthetic flux under HL should be regarded as provisional pending additional constraints on the model (for example, time-resolved multi-omics data or ^13^C metabolic flux analysis).

Purine metabolism showed a coordinated increase in several interconversion and biosynthetic reactions under LL, a pattern that would be consistent with accelerated nucleotide turnover as part of a light-limitation stress response, in line with reports linking purine catabolites such as allantoin to abiotic-stress signaling in plants. Two concerns apply to this interpretation. First, de novo purine biosynthesis carries a substantial ATP cost, and it is not immediately intuitive that an energy-limited condition would favor increased flux through ATP-consuming biosynthetic steps; a plausible resolution is that the observed flux reflects nucleotide turnover and salvage/interconversion reactions rather than net biosynthesis, although this cannot be distinguished with the present data. Second, examination of the underlying flux-difference distribution shows that a cluster of reactions, panning purine metabolism as well as several functionally unrelated reactions in glyoxylate and amino-acid metabolism, share a numerically identical HL–LL flux difference, a pattern more consistent with these reactions being tied to a single underlying network constraint than with independent regulation of each pathway. The purine-metabolism signal reported here should accordingly be treated as a model-generated hypothesis rather than a fully resolved metabolic shift, pending independent validation (for example, targeted profiling of purine and allantoin pools under HL and LL).

A predicted change in glyoxylate-cycle flux (isocitrate lyase, malate synthase) between HL and LL was also observed, although its direction was sensitive to the specific assumptions used to constrain light availability in the model and did not consistently meet the criterion for a robust, non-overlapping difference across the sensitivity analyses performed. The glyoxylate cycle is more commonly associated with acetate utilization, heterotrophic growth, or lipid mobilization in *C. reinhardtii* than with photoautotrophic metabolism, and a mechanistic link to light intensity is not established for this strain. Plausible, non-exclusive explanations include a connection to photorespiratory glycolate metabolism, an anaplerotic role specific to this natural strain, or an artefact of the wide flux bounds used to prevent iMAT from silencing central pathways. This result is presented as a preliminary, model-derived observation rather than a confirmed finding, and direct testing (for example, glyoxylate-cycle metabolite measurements or isotope tracing) would be needed before drawing a physiological interpretation.

Several transaminase reactions (tyrosine, cysteine/methionine, and alanine transaminases) showed higher flux under HL, broadly consistent with increased nitrogen assimilation and amino acid biosynthesis accompanying enhanced photosynthetic carbon fixation. A systematic, amino-acid-by-amino-acid analysis linking these changes to nitrogen-assimilation flux was beyond the scope of the present study. In addition, at least one of these reactions (alanine transaminase) belongs to the same numerically coupled cluster noted above for purine metabolism, so an independent, condition-specific regulatory signal cannot be confirmed for this pathway from the present analysis alone.

The *C. reinhardtii* CC-4414 has emerged as a particularly robust strain for studying light stress physiology due to its tolerance of high light intensities and its capacity for reactive oxygen species (ROS) management. Suwannachuen et al. (2023) [8] demonstrated that CC-4414 manages ROS somewhat more effectively than the sensitive strain CC-2344, with proteomic data revealing cellular modifications toward membrane proteins and the induction of palmelloid structures and cell aggregation that shield cells from excessive light. These morphological and physiological adaptations are broadly consistent with the increased flux predicted through photoprotective pathways under HL conditions in the present model. CC-4414 also displays notable metabolic plasticity and recovery capacity following chemical stress exposure, such as zinc oxide nanoparticle treatment, further indicating its resilience to environmental perturbation [7]. The strain-specific metabolic traits captured by *iYH2021* thus provide a more tailored representation of light stress responses than previous models based on laboratory reference strains, which typically lack the stress-tolerance traits of natural strains [13,14]. Future studies could extend this comparative approach to additional natural strains to identify core metabolic features underpinning stress tolerance across diverse genetic backgrounds [16,17].

The *iYH2021* model, together with the photon-handling and flux-redistribution patterns identified here, offers a starting point for guiding metabolic engineering strategies aimed at high-value bioproduct production under light stress. The elevated flux predicted through SBPase and the Calvin–Benson cycle under HL suggests this pathway as a candidate target for increasing production of pigments (e.g., carotenoids and chlorophylls) and metabolic precursors with pharmaceutical and functional-biomaterial relevance [17,18]. The parallel increase in predicted glycolytic and TCA-cycle flux under HL further suggests that carbon and energy metabolism are reorganized to support higher metabolic throughput under this condition, which, taken together with the higher theoretical growth rate estimated for HL (Section 3), points to CC-4414 as a candidate for large-scale outdoor cultivation in tropical, high-light environments and the photodamage-related concerns on realized growth discussed above. The model also provides a platform for exploring lipid and bio-hydrogen production pathways, which have been extensively studied in *C. reinhardtii* [4,14]. Incorporating additional condition-specific omics layers in future work may help refine predictions of metabolic bottlenecks and support strain-improvement strategies for bioproduct synthesis [9,10], and the addition of transcriptomic and metabolomic data would likely improve predictive accuracy and reveal further regulatory mechanisms underlying light-driven metabolic shifts [16,19].

This study has several limitations. The iMAT algorithm classifies reactions into high-, intermediate-, and low-expression categories using a threshold-based procedure that does not directly model the quantitative relationship between protein abundance and flux capacity; approaches that use enzyme abundance as a continuous constraint (e.g., E-Flux or GECKO-type formulations) may capture this relationship more faithfully and could be explored in future work. The 25th/75th-percentile thresholds used here are a widely adopted convention rather than a biologically validated cutoff for this dataset, and reactions lacking proteomic support were assigned the midpoint expression value, which may understate true condition-specific differences for those reactions. Flux variability analysis showed that a substantial fraction of the network is not uniquely constrained by the model and proteomics data at the optimum, so many of the flux differences discussed above are best regarded as consistent with, rather than proven by, the available data. Because the iMAT-derived context-specific networks could not support biomass synthesis without reintroducing most of the reactions removed by the algorithm, growth was not optimized directly on these networks; the separate growth-rate comparison presented in Section 3 used the unrestricted genome-scale network and should not be conflated with the proteomics-integrated flux analysis. This study also focused on steady-state light conditions, whereas natural and industrial cultivation environments experience dynamic light fluctuations; extending the model to dynamic light regimes and validating its predictions against time-resolved growth, metabolite, or flux data remain important directions for future work. Finally, direct experimental validation of the specific model predictions discussed above (purine-metabolism changes under LL, glyoxylate-cycle flux changes, and the HL/LL growth-rate estimate) was not performed in this study; these remain testable hypotheses generated by the model rather than established findings. We further note that the acetate and CO_2_ uptake bounds used in the supplementary growth-rate analysis (Section 2.4) are inherited default constraints from the iCre1355 template rather than experimentally measured, strain-specific physiological uptake rates for CC-4414, so the absolute growth-rate values reported should be treated as illustrative rather than validated predictions. We also reiterate that the maximum-abundance convention used for multi-subunit complexes (Section 4.6) is a practical simplification rather than a validated standard, and that light-uptake bounds are condition-appropriate model constraints rather than validated conversions of physical irradiance; both choices should be considered when interpreting the absolute flux values reported in this study.

This work contributes to the broader field of algal stress physiology by providing a systems-level understanding of how *C. reinhardtii* CC-4414 adjusts its carbon and energy metabolism in response to light stress. The reconstructed *iYH2021* model, which integrates strain-specific genomic and proteomic data, offers a robust predictive platform for studying metabolic adaptations under environmental stress. The identification of purine metabolism as a key stress-responsive pathway under LL conditions opens new avenues for investigating the role of purine-derived signaling molecules in microalgal stress physiology. Furthermore, the demonstration of enhanced photosynthetic and central metabolic fluxes under HL conditions provides a mechanistic basis for the superior biomass productivity of CC-4414 under high light intensities. From an applied perspective, these findings support the development of sustainable microalgal biorefineries by informing light regime optimization and metabolic engineering strategies for enhanced bioproduct synthesis. As the global demand for renewable energy and bio-based products continues to grow, strains such as *C. reinhardtii* CC-4414, combined with predictive systems biology tools like *iYH2021*, will play an increasingly important role in advancing the circular bioeconomy [20].

## 4. Materials and Methods

### 4.1. Sequence Retrieval and Quality Filtering

Whole-genome sequencing (WGS) data of *C. reinhardtii* CC-4414 were retrieved from the NCBI Sequence Read Archive (SRA) under accession number SRX4395102 (Run: SRR7526648). The dataset was generated using the Illumina HiSeq 2000 platform with paired-end sequencing, comprising 42.2 million reads and approximately 8.5 Gb of sequence data. Raw Illumina sequencing reads of *C. reinhardtii* CC-4414 were quality-filtered using FastP v0.23.4 [21] with the following parameters: average quality score ≥ Q30, read length ≥ 50 bp, and automatic adapter trimming.

### 4.2. De Novo Genome Assembly and Quality Assessment

The filtered reads were subsequently assembled de novo using SPAdes v4.0.0 with default parameters [22,23] with k-mer sizes of 21, 33, 55, 77, and 99, and all other parameters, including the coverage cutoff, left at default (automatic) settings. Assembly quality was evaluated using QUAST v5.3.0 [24]. Genome completeness was assessed with BUSCO v6.0.0 [25] using the chlorophyta_odb10 lineage dataset (n = 62 single-copy orthologs).

### 4.3. Gene Prediction and Protein Annotation

Gene prediction was performed on the assembled genome using AUGUSTUS v3.5.0 [26] with the *C. reinhardtii* species model and default parameters. Predicted protein sequences were then extracted from the annotation files using gffread v0.12.9 [27]. To minimize annotation artifacts and fragmented predictions, protein sequences shorter than 100 amino acids were removed using SeqKit v2.9.0 [28] prior to downstream functional annotation and metabolic network reconstruction.

### 4.4. Template-Based Metabolic Network Reconstruction

The genome-scale metabolic model of *C. reinhardtii* CC-4414 was reconstructed using a template-based approach. The published *iCre1355* model [13], which represents the most comprehensive and experimentally validated metabolic reconstruction for *C. reinhardtii* based on the JGI v5.5 reference genome [6], was selected as the template. Orthologous genes between *C. reinhardtii* CC-4414 and JGI v5.5 were identified using the best bidirectional hits (BBH) method via BLASTP, with a sequence identity threshold of ≥40% and an E-value cutoff of 1 × 10^−10^; bit score was used as a secondary criterion for selecting the best hit among candidates passing this filter. For genes without identifiable orthologs, manual curation was performed based on sequence homology and functional annotation, and for isozymes, the gene with the highest bit score was retained to represent the corresponding enzymatic reaction. Relative to the *iCre1355* template, this process, together with the gap-filling step described in Section 4.5, yielded a CC-4414-specific network in which 337 reactions were added and 3 template reactions (ATPM_NGAM, non-growth-associated ATP maintenance; INSH, inosine hydrolase; and TAT, L-tyrosine:2-oxoglutarate aminotransferase, consolidated with the retained isozyme reaction TYRTA) were not retained. The biomass objective function (Biomass_Chlamy_mixo, reflecting the mixotrophic, TAP-medium growth conditions used in this study) was inherited directly from *iCre1355* without modification to its stoichiometric composition, since strain-specific biomass composition and maintenance-energy data are not currently available for CC-4414; this reaction comprises 204 reactant and product terms, dominated by aminoacyl-tRNAs (protein synthesis), the four ribonucleoside and four deoxyribonucleoside triphosphates (RNA/DNA synthesis), photosynthetic pigments (chlorophyll a and b, β-carotene, lutein), membrane and storage lipids (mono- and digalactosyldiacylglycerol, sulfoquinovosyldiacylglycerol, phosphatidylethanolamine, phosphatidylglycerol, the betaine lipid DGTS, and a series of triacylglycerol species), and storage carbohydrate, with ATP and H_2_O consumed as the growth-associated maintenance (GAM) cost. The full model, including the biomass reaction, gap-filled reaction list, and reconstruction scripts, is available at https://github.com/pramotec/GSMN_chlamy (archived at https://doi.org/10.5281/zenodo.22767600) (see Data Availability Statement).

### 4.5. Gap-Filling of Incomplete Metabolic Pathways in the GSMN

Gap-filling decisions prioritized restoring pathway connectivity to the biomass objective while minimizing the addition of thermodynamically implausible or orphan reactions; every added reaction was individually cross-checked for mass and charge balance, as noted above. As noted in Section 4.4, an intermediate model (model_An.mat) corresponding to the network immediately prior to this gap-filling step is available and independently confirms that exactly 337 reactions were added at this stage to produce the final *iYH2021* model.

Gap-filling was performed to resolve incomplete metabolic pathways in the template-derived draft network. A candidate metabolic network for CC-4414 was independently generated using CarveMe v1.6.6 [29] from the annotated CC-4414 genome, and this CarveMe-derived network served as a source of candidate reactions not already present in the *iCre1355*-derived draft. Rather than incorporating all CarveMe-predicted reactions automatically, the CarveMe network was systematically compared against the draft model and candidate reactions were manually screened based on their reaction equation, EC number, gene associations, subsystem, and consistency with known *C. reinhardtii* metabolism; only reactions with adequate genomic and functional support were retained. Non-gene-associated reactions, including spontaneous and literature-based reactions, were curated and incorporated where required for pathway connectivity. Transport and exchange reactions were adjusted based on subcellular localization information to ensure network connectivity, and the network was further refined using the RAVEN Toolbox 2.0 [30]. Existing *iCre1355*-derived reactions were also manually reviewed for stoichiometry, reversibility, metabolite compartmentalization, and GPR associations; reactions lacking CC-4414 genome-annotation support, or judged redundant or inappropriate, were removed or modified. The KEGG database [31] was used to standardize GPR associations, reaction reversibility, and metabolite nomenclature, and all reactions were checked for mass and charge balance. Gap-filling decisions prioritized restoring pathway connectivity while minimizing the addition of thermodynamically implausible or orphan reactions. This process added 337 reactions to the draft network to produce the final *iYH2021* model (Section 4.4).

### 4.6. Integrating Proteomic Data in the GSMN

Mass spectrometry proteomics data were obtained from the ProteomeXchange (PXD040080) and JPOST (JPST002038) repositories [8]. These data correspond to *C. reinhardtii* CC-4414 cultures grown in Tris-Acetate-Phosphate (TAP) medium at 25 °C under ambient CO_2_ and continuous illumination in liquid shake culture (180 rpm), with log-phase cultures diluted to 2 × 10^6^ cells mL^−1^ before the shift to high light; HL samples were collected 2 days after this shift (1500 µmol photons m^−2^ s^−1^), and LL samples were collected from cultures maintained continuously at 50 µmol photons m^−2^ s^−1^, both delivered by white LED lamps and measured with an LI-250A Light Meter (LI-COR Biosciences, Lincoln, NE, USA) [8]. For each condition, n = 3 independent biological replicates were cultured and pooled prior to protein extraction, and the pooled sample was analyzed by LC-MS/MS with n = 3 technical replicate injections [8].

Proteomic data were integrated into the genome-scale metabolic model using the iMAT algorithm [15] implemented in the COBRA Toolbox [32]. Protein abundance values were mapped to corresponding reactions via gene-protein-reaction (GPR) associations; for reactions catalyzed by multiple gene products, the maximum abundance value among the corresponding proteins was retained. We note that, because the activity of a multi-subunit complex is generally constrained by its least abundant essential subunit, this maximum-value convention may overestimate effective complex activity in some cases; it was adopted here as a practical approximation rather than a validated standard, and its potential influence on the resulting flux predictions is acknowledged as a limitation (Section 3). Protein abundance values were log_2_-transformed (log_2_[x + 1]) to normalize the distribution, and the 25th and 75th percentiles of the log-transformed expression values were calculated independently for the HL and LL conditions to define low- and high-expression thresholds, respectively, following the approach of Zur et al. [15] (HL: 25th percentile = 17.12, 75th percentile = 19.88 log_2_ units; LL: 25th percentile = 16.97, 75th percentile = 18.53 log_2_ units; computed from the n = 172 proteins with proteomic support). Reactions lacking proteomic support were assigned the midpoint value of the corresponding thresholds. To prevent excessive pruning of central pathways by the threshold-based procedure, reactions in photosynthesis, the Calvin–Benson cycle, glycolysis, the TCA cycle, and the biomass and photon-handling reactions (Biomass_Chlamy_auto, Biomass_Chlamy_mixo, PRISM_design_growth, PSIred, PSIIred, CBFC, FNORh, ATPSh, HCO3Ehi, RBPCh, PRUK, GAPD, PGK, ICDH, PDH, CS, FBA3hi, FBA4hi, and TPI) were assigned wide flux bounds (−1000 to 1000 mmol gDW^−1^ h^−1^) prior to context-specific network reconstruction. Context-specific metabolic networks for the HL and LL conditions were then reconstructed using the iMAT algorithm. Flux balance analysis (FBA) was therefore performed on each context-specific network using PRISM_design_growth—a reaction that partitions visible-photon uptake (EX_photonVis_e) into wavelength-specific photon pools (photonVis_e → 0.22079 photon646_h + 0.63834 photon673_u + 0.73362 photon680_u)—as the objective function, so that the resulting flux distributions reflect photon-handling capacity and internal flux redistribution between conditions. Light uptake (EX_photonVis_e) was constrained to −1000 mmol gDW^−1^ h^−1^ for the HL model, a value chosen to represent non-limiting light supply based on a separate sensitivity analysis (Section 2.4) showing that predicted growth on the unrestricted network plateaus once photon uptake exceeds approximately −67 in the same flux units. For the LL model, this bound was set to −50, numerically matching the LL light intensity (50 µmol photons m^−2^ s^−1^) reported in the source proteomics study [8]. We emphasize that this photon-exchange flux unit has not been validated as a direct, quantitative conversion of physical irradiance: no photon absorption cross-section, quantum yield, or other conversion factor was applied, and the HL bound (−1000) does not correspond numerically to the experimental HL intensity of 1500 µmol photons m^−2^ s^−1^. These model bounds should therefore be understood as condition-appropriate constraints, chosen using the sensitivity analysis and the numerical LL intensity as a reference point, rather than as validated physical unit conversions. All computations were performed in MATLABR2026a (Version 26.1) (The MathWorks, Inc., Natick, MA, USA) using the COBRA Toolbox [32] with the Gurobi Optimizer v13.0.1 (Gurobi Optimization, LLC, Beaverton, OR, USA). The resulting flux distributions were compared to identify differential metabolic activities between HL and LL conditions, and flux variability analysis (FVA, fluxVariability() in the COBRA Toolbox, 100% of optimum) was performed on each context-specific model to assess the robustness of these differences (Section 2.3.3).

### 4.7. Supplementary Theoretical Growth-Rate Analysis

Since the iMAT-derived context-specific models are not compatible with biomass optimization (Section 3), a separate analysis was performed to estimate growth capacity directly, using the unrestricted genome-scale network (prior to iMAT-based context-specific restriction) with Biomass_Chlamy_mixo as the objective. The visible-photon exchange reaction (EX_photonVis_e) was bounded at the same HL and LL light capacities used in Section 4.6 (−1000 and −50, respectively) as the sole condition-specific constraint, providing an estimate of the network’s theoretical maximum growth capacity under each light condition, independent of the proteomics/iMAT integration (results in Section 2.4). To characterize the constraints on predicted growth more generally, a systematic sensitivity screen was performed on the same unrestricted network: photon uptake was varied to identify the point beyond which additional light no longer increased predicted growth, and every exchange reaction was individually tested by doubling its uptake bound and re-optimizing biomass to identify which substrates most strongly limited growth. A two-dimensional sensitivity analysis was then performed over acetate (EX_ac_e) and CO_2_ (EX_co2_e) uptake bounds (−2 to −4.5 mmol gDW^−1^ h^−1^ for each), with photon uptake fixed at −80 to ensure light was not limiting, and biomass flux recorded for each combination of bounds (results in Section 2.4).

## 5. Conclusions

In this study, we successfully reconstructed a genome-scale metabolic model of *C. reinhardtii* CC-4414, designated *iYH2021*, through the integration of *de novo* genome assembly and quantitative proteomics. The model comprises 2021 genes, 2601 reactions, and 2089 metabolites, representing the first genome-scale metabolic reconstruction reported for this light-tolerant natural strain. Flux variability analysis identified a small subset of reactions, most notably phosphoglycerate kinase, whose condition-specific flux differences between high-light (HL) and low-light (LL) conditions are robust to solution non-uniqueness; these represent the most defensible, high-confidence outcomes of the flux-redistribution analysis and offer concrete targets for future mechanistic and experimental follow-up. The broader point-solution patterns observed across the network—including apparent flux enhancement through photosynthetic electron transport, the Calvin–Benson cycle, glycolysis, and the TCA cycle under HL, and upregulation of purine metabolism under LL—did not meet this robustness criterion for most individually examined reactions and should accordingly be regarded as candidate hypotheses generated by the model rather than confirmed evidence of condition-specific regulation. A complementary, theoretical growth-rate analysis using the unrestricted network additionally indicated a higher maximum growth rate under HL than LL. Together, these results illustrate both the metabolic plasticity of *C. reinhardtii* CC-4414 and its capacity to rewire carbon and energy allocation in response to fluctuating light environments. The *iYH2021* model, together with its associated data and code, provides a transparent platform for future studies of metabolic engineering strategies for high-value bioproduct production under industrial light regimes. This work thus contributes to the broader field of algal stress physiology and supports the development of sustainable microalgal biorefineries for the circular bioeconomy. We reiterate that the flux-based findings summarized above are model-derived predictions that await independent experimental validation.

## Figures and Tables

**Table 1:** Comparison of genomic characteristics between *C. reinhardtii* CC-4414 and the canonical reference strain.

Genome Features	*C. reinhardtii* (Wild Type) [6]	*C. reinhardtii* CC-4414 (In This Study)
Assembly Length (Mb)	121 Mb	94.02 Mb
G + C Content (%)	64.00%	64.30%
Total Predicted Proteins	15,143	16,838

**Table 2:** Comparison of components in *iCre1355* and *iYH2021.*

Components	*iCre1355* [13]	*iYH2021* (In This Study)
Genes	1355	2021
Genome version	JGI version 5.5	NCBI (SRX4395102) *
Reactions	2394	2601
Compartment	10	10
Metabolites	1845	2089

* Note: SRX4395102/SRR7526648 are NCBI Sequence Read Archive (SRA) accessions for the raw sequencing reads only.

**Table 3:** Comparison of representative metabolic pathways and reaction trends under HL and LL conditions.

Pathway	HL vs. LL Trend	Representative Reactions (Examples)	Biological Interpretation
Photosynthesis	HL significantly enhanced	PSIred, CBFC, FNORh, PSIIred, CEF, ATPSh	Under high light, Photosystem I/II, cytochrome b6/f complex, and cyclic electron flow are enhanced, producing more ATP and NADPH.
Carbon Fixation (Calvin Cycle)	HL significantly enhanced	RBPCh, PRUK, FBA3hi, SBP, HCO3Ehi	Higher CO_2_ fixation capacity, supporting faster biomass synthesis.
Glycolysis/Gluconeogenesis	HL enhanced	PYK, ENO, PGM, PGK	Faster conversion of photosynthetic products into energy and precursors, supporting growth.
TCA Cycle and Oxidative Phosphorylation	HL partially enhanced	CS, FUMm, CYOO6m, ATPSm	Higher energy demand under high light leads to increased respiratory chain activity.
Glyoxylate Cycle	HL enhanced	ICL, MALSm	May help balance carbon metabolism.
Purine Metabolism	LL relatively enhanced	Multiple PUNP, IMPD, PRFGS, etc.	Under low light, more nucleotide turnover or stress response may be required.
Amino Acid Metabolism	Mixed (HL stronger in some transaminases)	TYRTA, CYSTAm, ALAAT	Amino acid synthesis and conversion are more active under high light.

**Table 4:** Flux variability analysis (FVA, 100% of optimum) of the HL- and LL-constrained context-specific models, performed using COBRA Toolbox’s fluxVariability() with the Gurobi solver. All values in mmol gDW^−1^ h^−1^.

Reaction	HL min	HL max	LL min	LL max	Ranges Overlap?
**ALAAT**	−1000	1000	−1000	1000	Yes
**ATPSh**	0	27	7.98	7.98	Yes
**ATPSm**	0	13.33	6.34	8.93	Yes
**CBFC**	0	27	15.96	15.96	Yes
**CS**	0	1	0.11	1	Yes
**CYOO6m**	0	10	4.75	6.69	Yes
**ENO**	0	32	22.77	29.24	Yes
**FNORh**	0	27	15.96	15.96	Yes
**FUMm**	0	1	0.11	1	Yes
**ICL**	0	1	0.11	1	Yes
**MALSm**	0	1	0.11	1	Yes
**PGK**	0	6	22.77	30.53	No
**PGM**	−1	0	−1	−0.11	Yes
**PRISM_design_growth**	1000	1000	50	50	No
**PRUK**	−1	0	−1	−0.11	Yes
**PSIIred**	0	13.50	7.98	7.98	Yes
**PSIred**	0	54	15.96	15.96	Yes
**PUNP1_1**	−1000	1000	−1000	1000	Yes
**PYK**	0	32	22.77	29.24	Yes
**RBPCh**	−1	0	−1	−0.11	Yes
**TYRTA**	−1000	1000	−1000	1000	Yes

## Data Availability

The whole-genome sequencing data for *C. reinhardtii* CC-4414 used in this study are available from the NCBI Sequence Read Archive (SRA) under accession number SRX4395102 (Run: SRR7526648). The mass spectrometry proteomics data are available from the ProteomeXchange consortium (PXD040080) and JPOST (JPST002038) repositories. The genome-scale metabolic model *iYH2021* reconstructed in this study is deposited in a persistent public repository (https://github.com/pramotec/GSMN_chlamy), archived on Zenodo with the persistent identifier https://doi.org/10.5281/zenodo.22767600, together with the associated flux-analysis code and input data, to ensure reproducibility. All other data generated or analyzed during this study are included in this published article.
